# Transverse testicular ectopia in a newborn with transposition of the great arteries: A unique case report

**DOI:** 10.1016/j.ijscr.2024.110347

**Published:** 2024-09-25

**Authors:** Minella Lalloz, Marilyn Wong, Roy Kimble

**Affiliations:** a The Queensland Children's Hospital, 501 Stanley Street, South Brisbane, QLD 4101, Australia; b The University of Queensland, St Lucia, QLD 4072, Australia

**Keywords:** Transverse testicular ectopia, Persistent Mullerian duct syndrome, Transposition of the great arteries, Variations in sexual characteristics, Case report

## Abstract

**Introduction:**

Transverse testicular ectopia (TTE) is an extremely rare though well-documented congenital anomaly. In males with a 46XY karyotype, it is characterised by the herniation of both testes and part of the Müllerian organs into a single processus vaginalis. TTE is one of the three main clinical presentations of persistent Müllerian duct syndrome (PMDS). Transposition of the great arteries (TGA) is another rare congenital anomaly and severe cardiac condition. We present the likely first reported case of TTE with an accompanying malformation of TGA in a newborn.

**Case presentation:**

A 3-day-old Caucasian 46XY newborn with TGA was referred to the paediatric surgeons and endocrinologists for possible variations of sex characteristics (VSC). Despite a clinical examination revealing phenotypical male genitalia, an early postnatal ultrasound (US) suggestive of a uterine structure raised the suspicion of VSC.

This patient had an arterial switch operation at 2 weeks of age before undergoing an exploration of the left groin at 8 weeks of age. Intraoperative findings revealed bilateral testes either side of a rudimentary uterus with fallopian tubes in the left inguinal canal. To avoid de-vascularising any structures, modified bilateral orchidopexy was performed placing each testis in the respective hemiscrotum with the uterus placed across the scrotal septum.

**Conclusion:**

We present the first reported case of TGA accompanying TTE. Early and accurate diagnosis, combined with the coordinated care by the specialist paediatric surgeon, cardiothoracic team, endocrinologist, and radiologist are essential for delivering timely, optimal care. This unique case raises the possibility of there being a link between TTE and TGA.

## Highlights

• Transverse testicular ectopia (TTE) is an extremely rare congenital anomaly
• TTE is one of three main clinical presentations of persistent müllerian duct syndrome (PMDS)
• Although TTE is rare, it is well-documented in the literature. However, this is likely the first reported case of TTE in a newborn with TGA, which raises the possibility of there being a link between these two congenital anomalies

## 1 Introduction

Transverse testicular ectopia (TTE), also known as crossed testicular ectopia, is one of the three main clinical presentations of persistent Müllerian duct syndrome (PMDS), along with bilateral cryptorchidism and unilateral cryptorchidism. TTE is an extremely rare congenital anomaly in which both testes and part of the Müllerian organs – including the fallopian tubes, uterus, and upper vagina – have herniated into a single processus vaginalis in males with a 46XY karyotype [1,2]. TTE may be classified under the broad umbrella term of variations of sex characteristics (VSC) or intersex variations. Formerly known as ‘disorders of sex development’, VSC or intersex variations have been increasingly adopted non-discriminatory terms to describe ‘congenital conditions in which development of chromosomal, gonadal, or anatomical sex is atypical’ [3,4].

Transposition of the great arteries (TGA) is a severe congenital cardiac malformation resulting from an embryological discordance between the aorta and pulmonary trunk [5]. Consequently, the aorta arises from the morphological right ventricle, whilst the pulmonary artery arises from the morphological left ventricle. TGA is a congenital anomaly accounting for 3 % of all congenital heart diseases and 20 % of the cyanotic heart diseases [6,7].

Although TTE is a rare entity, with an estimated incidence of 1 in every 4 million children [8], it is well-defined in the literature. However, we present the likely first reported case of TTE co-occurring with TGA in a newborn treated in Australia. Here we discuss the diagnostic challenges encountered, including the need to obtain parental gene panel testing, and the importance of early and accurate identification of both conditions for optimal treatment and prognosis. Moreover, this case highlights the crucial role of the multidisciplinary team (paediatric surgeons, the cardiothoracic team, endocrinologists, and radiologists) in providing comprehensive guidance when managing similar cases. We believe this unique case of TTE with accompanying TGA will add new and valuable knowledge to the medical literature by emphasising the complexity of such a presentation and the importance of adequate counselling, follow up surveillance and ongoing management.

This work has been reported in line with the SCARE criteria [9].

## 2 Case presentation

We present a 3-day-old Caucasian newborn with phenotypical male genitalia (penile tissue and empty scrotum) referred to the paediatric surgical team and endocrinologists for possible VSC. This was prompted following an early postnatal ultrasound (US) scan reporting the presence of a uterine structure and no evidence of gonads in the pelvis.

Significant medical history included an antenatal diagnosis of TGA with intact ventricular septum for which the patient underwent a balloon atrial septostomy day 1 of life. This patient was awaiting an arterial switch operation for definitive treatment of the cardiac malformation. Current medications pre-operatively were prostin, paracetamol and supplemental vitamins. A maternal history was also obtained and revealed iron-deficiency anaemia, gestational diabetes, and pregnancy-induced hypertension with an otherwise unremarkable antenatal course. There was no significant paternal medical history. Clinical examination of the patient revealed a normal-sized phallus and anatomical position of the urethra on the glans, a palpable gonad in the left groin and fused labioscrotal folds. No gonads were appreciable in the scrotum.

**Fig. 1:**
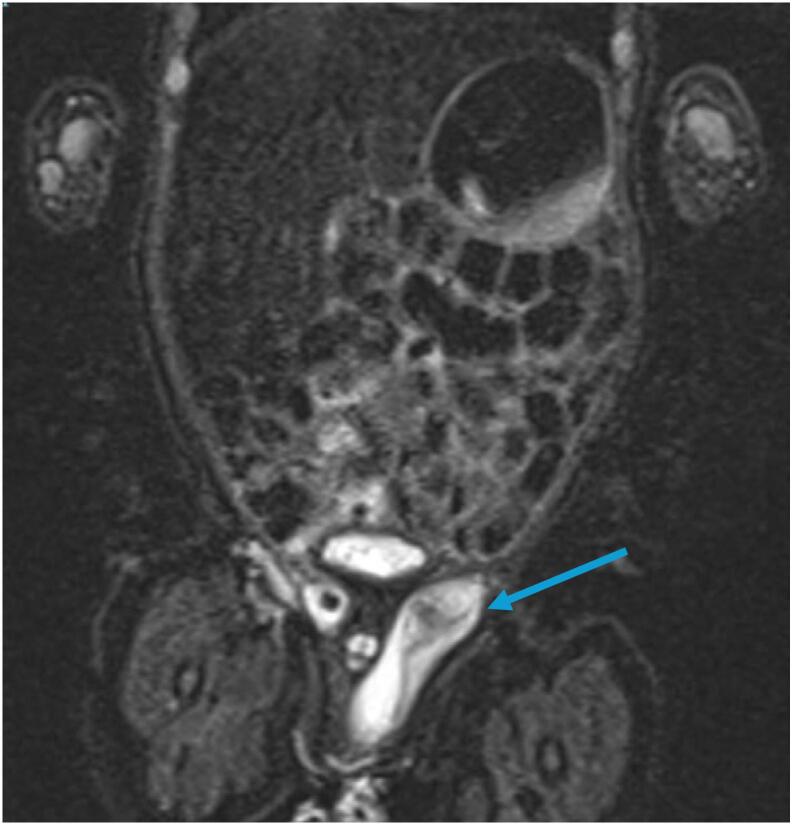
(Right) A coronal section of the abdominopelvic MRI scan demonstrating a left indirect inguinal hernia (blue arrow) initially reported as a left-sided scrotal sac containing small non-dilated bowel loops proximally and possible undescended left testis. Indication for the scan was to investigate for possible VSC in a 1-week-old phenotypical male with TGA. Clinical history included a normal penis, palpable gonad in the left groin and empty scrotum on examination combined with a uterine structure seen on an early postnatal US scan. Of note, no female genitalia (uterus or ovaries) were identified on this MRI. (For interpretation of the references to colour in this figure legend, the reader is referred to the web version of this article.)

**Fig. 2:**
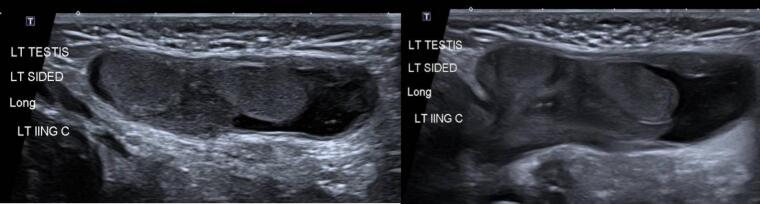
(Below) Interval US scan of a 2-week-old phenotypical male being worked up for likely VSC. US demonstrates a left indirect sliding inguinal hernia containing two adjacent rounded hypo-isoechoic structures (13 × 5 × 8 mm and 7 × 10 × 9 mm), presumed to be gonads.

Subsequent abdominopelvic imaging was undertaken to further evaluate the presence of male or female gonads. A magnetic resonance imaging (MRI) scan (Fig. 1) revealed two contradictory findings: firstly, a left-sided scrotal sac containing small non-dilated bowel loops proximally, and secondly, a possible undescended testis in the left inguinal canal with no evidence of female genitalia, like the uterus or ovaries. A right testis and scrotal sac were also absent. Given the incongruence in results between the early postnatal US scan and those of the MRI, the decision was made to organise another abdominopelvic US (Fig. 2). Fortunately, the repeat US clearly demonstrated a sliding left inguinal hernia containing two presumed gonads. It is also worth noting that the visualised liver, spleen, kidneys, and adrenal glands appeared normal on both imaging modalities. A second review of the MRI was requested and it was confirmed that these images were indicative of a left indirect inguinal hernia likely containing at least one testis, though again, no uterine structure was observed.

Overall, the clinical examination findings of an empty right hemiscrotum and palpable left groin lump were able to be correlated with the US findings of two gonads in the left inguinal canal: hence, in keeping with the diagnosis of TTE.

## 3 Therapeutic intervention

This patient was discussed in a multidisciplinary team setting to establish consensus on gonadal anatomy and ongoing management with regards to the concurrent TGA. Expert opinions from paediatric general and cardiothoracic surgeons, endocrinologists and radiologists were therefore obtained. On review of the karyotyping, it was confirmed that the patient is genetically male, 46XY. A screen for DiGeorge syndrome (22q11.2 deletion) – a condition featuring conotruncal cardiac anomalies, hypocalcaemia, palate defects and less commonly, genital anomalies [10] – returned negative. Both AMH and inhibin B levels were normal, indicating a normal hypothalamic-pituitary-gonadal axis. Testosterone levels were also normal, indicating the presence of Leydig cells. When combining these blood test results with that of the serial US scans and clinical examination findings, it was agreed that the left indirect inguinal hernia likely contained two gonads, presumably testes, which was most in keeping with overall diagnosis of TTE.

This patient underwent an arterial switch operation with the cardiothoracic surgeons at 2 weeks of age. This procedure involved a median sternotomy, excision of the thymus, division of the patent ductus arteriosus, transection of the great vessels with transfer of the coronary arteries as buttons of tissue to the neoaortic root, and closure of the atrial septostomy. Intraoperative transthoracic echocardiography was satisfactory and demonstrated good biventricular function. A total of three drains were inserted: one mediastinal, one right pleural and one peritoneal drain. The sternum was initially left open – with simple prolene sutures to the skin and dressing - before being formally closed 3 days later.

**Fig. 3:**
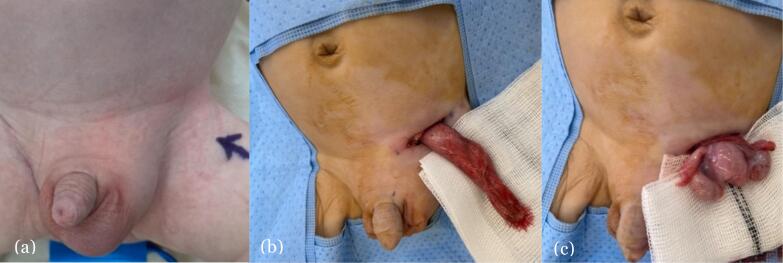
(Below) Intraoperative clinical photographs of the left inguinoscrotal exploration in an 8-week-old phenotypical male with likely VSC: (a) Visible left inguinoscrotal swelling secondary to the indirect inguinal hernia and an empty right hemiscrotum. Normal appearing external male genitalia (b) Delivery of the left inguinal sac through the inguinal incision containing the two gonads identified on US imaging (c) Opening of the left patent processus vaginalis revealing two gonads with the accompanying uterus and fallopian tubes. These findings are most consistent with the diagnosis of TTE, which is one of the three main clinical presentations of PMDS.

After an uneventful recovery from cardiac surgery, the paediatric surgeons performed a left groin exploration at 8 weeks of age. Through a left groin incision and inguinal dissection, the patent processus vaginalis (PPV) was identified and delivered via the wound. This was then opened to reveal the right gonad in the inguinal canal and the left gonad emerging through the internal ring. Between said gonads lay a rudimentary uterus and fallopian tubes with likely vas deferens running laterally on either side (Fig. 3). These findings are consistent with TTE. To avoid de-vascularising any structures, careful dissection to separate the vascular pedicles, fallopian tubes, and uterus complex from the PPV was performed with minimal difficulty. A modified bilateral orchidopexy was undertaken by placing each testis in the respective hemiscrotum with the uterus placed across the scrotal septum. The decision to preserve both testes and rudimentary uterus was in order to reduce the risk of damage to the existing gonads.

It must also be mentioned that both gonads had a heterogenous appearance to the upper pole region, hence, biopsies of each were obtained. Histopathology revealed left and right gonad immature testis with an area of testicular dysgenesis. No gonadoblastoma nor other germ cell tumour was identified.

Gene panel testing of the parents was carried out following extensive counselling on TTE and after obtaining informed consent. Both the parents were identified as carriers of the AMHR2 missense variant, accounting for the PMDS observed in their child.

At the 12-month review, this patient was found to be a healthy 1-year old toddler with a stable cardiac function and no complications post modified bilateral orchidopexy. Annual surveillance from a heart perspective and continued assessment of the testes for any malignant transformation and fertility issues remains ongoing through serial scrotal US and clinical examinations.

## 4 Clinical discussion

In males, sex differentiation is primarily driven by two hormones: anti-Mullerian hormone (AMH) and testosterone. Persistent Mullerian duct syndrome (PMDS) is typified by AMH either not being secreted or being inactive, which occurs as either a result of mutations in the activation of AMH or its receptor, AMHR2 [1].

TTE can be classified into 3 types; type 1 is associated with inguinal hernia only (50 % of cases), type 2 is associated with PMDS (30 % of cases) and type 3 is associated with genitourinary abnormalities (20 % of cases), such as hypospadias. This case of TTE belongs to the type 2 category, which is typified by the presence of uterus and fallopian tubes in an otherwise phenotypically normal 46XY male [[11], [12], [13]].

This patient is a firstborn child with TTE and PMDS due to compound heterozygous AMHR2 gene mutation. Specifically, both parents were identified as carriers of the AMHR2 missense variant, with one being found to be a carrier of the familial c.1046 T > C (p. (Ile349Thr) AMHR2 variant, and the other being a carrier of the familial c.1240G > A (p. (Ala414Thr) AMHR2 variant. PMDS is an inherited disease transmitted as an autosomal recessive trait. As such, the probability of this couple having a child with both AMHR2 variants is 1 in 4, and appropriate genetic counselling offering insights into the recurrence risk for future pregnancies and implications for family planning would be highly encouraged.

To date, a total of 59 AMHR2 gene mutations have been described, including 36 missense, 11 stop, 8 deletions and 4 splicing defects [1,11,14]. Patients with mutations in AMHR2 gene present fully virilised with male secondary characteristics. The presence of a uterus is usually detected early in childhood due to cryptorchidism (undescended testis) and associated hernias [11,15]. TTE occurs in about 25 % of patients with AMH or AMHR2 mutations [1]. The most common complication of PMDS is infertility, with reports of less than 20 % of patients with AMH or AMHR2 mutations having successfully reproduced [1]. Moreover, early orchidopexy for unilateral or bilateral cryptorchidism is recommended as there is documented evidence of malignant testicular degeneration occurring in 33 % of patients with PMDS from 18 years of age [1]. Although very rare, malignant transformation of Mullerian derivates has also been described [16].

In stark contrast, a current survey of the literature shows there are no reports published concerning TGA accompanying mutations of the AMHR2 gene. Similarly, there are no links described between TTE and TGA, and therefore the possible mechanisms remain unknown. The role for gene panel testing, however, remains important to deepen our knowledge of PMDS and TTE.

Final valuable lessons to take from this case are to emphasise and offer early, appropriate counselling of the family, in terms of planning for future children, and regarding the gender identity of the child in question. It is important to discuss the probability (1 in 4) of having another child with PMDS secondary to compound heterozygous AMHR2 gene mutation and the implications of this, including the risks of infertility and malignant testicular or Mullerian derivate transformation. In a genotypical and phenotypical male living with Mullerian remnants (in this case, a uterus and fallopian tubes), providing adequate psychological support is essential to promoting a state of physical, emotional, and social wellbeing in relation to sexuality. This is particularly important for both the patient and their family members who may aid in navigating the potential challenges of gender identity and VSC. Substantial support is even more so relevant for patients living with the long-term effects following corrective cardiac surgery for TGA, which itself may leave significant physical and emotional scars along with the burden of cardiovascular and cardiothoracic reviews and life-long medications.

## 5 Conclusion

This is a unique and likely first reported case of TTE with accompanying TGA in a newborn. This adds to the current literature on both TTE and TGA as well as provides insight into a potential link between said conditions. Early identification and coordinated care by the specialist paediatric surgeon, cardiothoracic team, endocrinologist, and radiologist are essential to best counsel the parents on the diagnoses and recommended management of these complex conditions. Whether a link exists between these two congenital anomalies remains uncertain given the lack of current evidence. Therefore, ongoing documentation of congenital cardiac anomalies, such as TGA, occurring with PMDS or specifically TTE, should be encouraged.

## Consent

Written informed consent was obtained from the patient's parents for publication and any accompanying images. A copy of the written consent is available for review by the Editor-in-Chief of this journal on request.

## Ethical approval

Ethics approval is not required for case reports deemed not to constitute research at the Queensland Children's Hospital. This case report was therefore exempt from Children's Health Queensland (CHQ) Human Research Ethics Committee (HREC) review.

## Funding

No funding nor grant support was provided.

## Author contribution

First author (myself, Dr. Minella Lalloz) – (1) obtaining written informed consent from the parents of the patient (a minor) described in this case report, (2) writing the paper and (3) collection of the relevant radiological and clinical images.

Second author (Dr Marilyn Wong) – editing of the case report.

Third author (Professor Roy Kimble) – editing of the case report.

## Guarantor

Roy Kimble.

## Research registration number

N/A.

## Conflict of interest statement

All authors declare there are no financial nor personal conflicts of interest to disclose.
